# Salvaging the Esophagus: Endoscopic Success After Damage-Control Surgery for Boerhaave Syndrome

**DOI:** 10.1016/j.atssr.2025.07.010

**Published:** 2025-07-31

**Authors:** Helena Bugacov, Cristina Cusmai, Tian Sun, Satish Nagula, Raja Flores, Daniel Laskey

**Affiliations:** 1 Department of Medical Education, Icahn School of Medicine at Mount Sinai, New York, New York; 2 Department of Thoracic Surgery, Icahn School of Medicine at Mount Sinai, New York, New York; 3 Department of Gastroenterology, Icahn School of Medicine at Mount Sinai, New York, New York

## Abstract

Boerhaave syndrome is a life-threatening condition often requiring emergency surgery, but recent endoscopic advancements offer alternatives. We present a case of esophageal perforation in a 58-year-old man managed with damage-control esophageal exclusion surgery, followed by luminal salvage with endoscopic stenting. Surgery revealed a large distal esophageal tear, which was excluded from a stapling device. During endoscopic evaluation, a wire was passed through the staple line, thus allowing stent deployment and restoring patency. The stent was later removed, with complete tissue recovery. This case highlights the potential of advanced endoscopic therapies to manage esophageal perforations and reduce the need for surgical procedures.

Boerhaave syndrome, esophageal perforation secondary to aggressive vomiting, is a rare and life-threatening condition. This syndrome has traditionally been considered a surgical emergency because of the high morbidity and mortality associated with the related mediastinitis. Surgical options include primary repair vs exclusion and esophageal diversion, depending on the timing of presentation and the patient’s clinical condition. However, endoscopic approaches are now challenging this convention for the treatment of contained perforations.^1^ Endoscopy is a less invasive management option, and any subsequent required surgery is often a thoracic washout with lower associated morbidity. Here we describe a case of acute esophageal perforation initially treated by emergency damage-control surgical esophageal exclusion with subsequent luminal recannulation using endoscopic stent placement.

The patient was a 58-year-old man with a past medical history of rheumatoid arthritis treated with prednisone, prostate cancer, substance use and alcohol disorder, gastroesophageal reflux disease, and a distal esophageal stricture, for which he previously underwent endoscopic dilation twice in the past 2 years. He initially presented to the emergency department of an outside hospital with worsening chest and abdominal pain after multiple episodes of forceful emesis several hours earlier. Pneumomediastinum on imaging was concerning for distal esophageal perforation, and he was taken on an emergency basis to the operating room. A distal esophageal injury involving 75% of the esophagus circumference was found proximal to the gastroesophageal junction with mucosal dissection into the proximal stomach, with extensive inflammation and concern for necrosis.

Given the patient’s extensive injury, the decision was made to perform a damage-control esophageal exclusion with a transabdominal stapler, both proximal to the perforation and distal to the gastroesophageal junction on the proximal stomach. A venting gastrostomy tube was placed (Figure). The plan was for a second-stage esophagectomy when he was clinically stable; however, he continued to have persistent sepsis, resulting in his transfer to our tertiary care center. Source control of bilateral pleural effusions was achieved with tube thoracostomies. Given his persistent sepsis, an esophagram was performed that demonstrated a persistent leak from the proximal esophageal stump. This was followed by an upper endoscopy, where he was found to have a pinhole defect in the proximal esophageal staple line. There was brisk extravasation of contrast material into the left side of the chest on endoscopic-guided fluoroscopy; however, contrast material also was noted to cross a defect in the staple line and fill the stomach. There was a small pinhole in the proximal staple line contrast injection demonstrated a small, tortuous tract across the distal staple line into the stomach, as well as extravasation into the pleural space. We advanced a guidewire through this tortuous tract into the stomach, and the guidewire allowed us to deploy a stent safely across the staple lines, thereby occluding the leak site and recanalizing the exclusion. A wire was able to traverse through this defect into the stomach, and a covered metal 18 mm × 120 mm stent (fully covered Agile, Boston Scientific) was deployed. In this case, the distal esophagus exhibited angulation and tortuosity across both staple lines. These anatomic challenges allowed for the more flexible Agile stent to be navigated.

**Figure:**
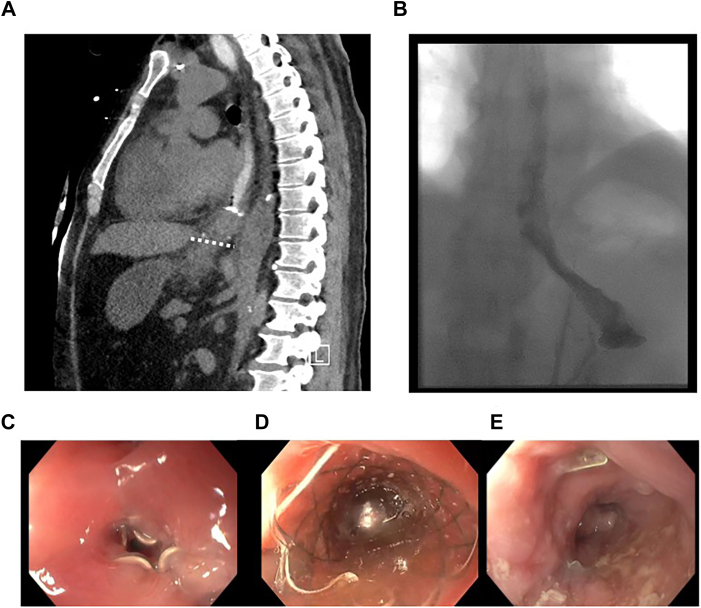
(A) Computed tomographic scan after emergency surgery with staples (dotted line demarcates placement of distal staples). (B) Esophagram showing restoration of flow after successful stenting. (C) Endoscopy with an exclusion staple line. (D) Endoscopy showing stent deployment through the staples. (E) Endoscopy after removal of the stent showing resolved and healed esophagus.

A postprocedure esophagram confirmed luminal patency without further extravasation (Figure). The patient went on to make a full recovery. The stent was removed 2 months after the procedure, with a well-healed site of perforation and no evidence of stenosis (Figure B). Seven months postoperatively, he reports some dysphagia but overall is better and able to tolerate a normal diet. His reflux has improved. The follow-up evaluation showed mild narrowing of the distal esophagus consistent with his preexisting stricture, for which he is currently undergoing endoscopic dilation, with improvement in symptoms.

## Comment

Boerhaave syndrome has morbidity and mortality rates as high as 50 % and is a critical surgical emergency.^2^ Emergency surgery is the mainstay of treatment, with options ranging from primary repair to esophageal exclusion with esophagostomy diversion, depending on the timing of presentation and patient’s clinical status.^3^^,^^4^ Primary repair is the desired outcome; however, damage-control diversion with cervical esophagostomy may be the only appropriate option. A delayed second-stage reconstruction is possible, although its feasibility is dependent on the clinical scenario and is often challenging. In the 1990s, some investigators suggested temporary esophageal exclusion using linear stapling devices with spontaneous recannulation. However, this technique is not well described in the literature and did not catch on. In this patient with a recalcitrant esophageal stricture, consideration could have been given to performing an esophagectomy, usually a preferred option for patients with refractory strictures and extensive esophageal disease.^5^ However, his tenuous clinical status prevented this from being a safe option. Instead, endoscopic therapy allowed us to gain immediate source control and defer definitive management of the stricture until after his sepsis had resolved.

Advances in therapeutic endoscopy have made nonsurgical approaches possible. Techniques such as endoscopic clipping (both through the scope and over the scope), endoluminal suturing device use, endoscopic vacuum therapy, and esophageal stenting facilitate salvage of the native esophagus and can avoid morbidity.^6^ Currently, one-half of esophageal perforations can be treated nonoperatively.^7^ Typically, this approach is reserved for contained perforations in a clinically stable patient. This case pushes the boundaries of nonoperative management of esophageal perforation. Our patient avoided having either a highly morbid, complex, second-stage esophagectomy or a cervical esophagostomy in a hostile surgical field. Although this case presentation may be a rare application for endoscopic intervention, unlikely to be seen often clinically, it represents the capabilities possible with a nonoperative technique. It shows that endoscopic intervention can be applied to more than stable, well-controlled, contained leaks and that in the right scenario, with close clinical monitoring, and a well-considered threshold to take for surgery, an endoscopic approach to advanced and complex esophageal perforation is possible, even in the presence of sepsis.
